# Supplementary material for: Mapping complex cell morphology in the grey matter with double diffusion encoding MR: A simulation study
Source: Neuroimage. 2021 Nov 1;241:118424. doi: 10.1016/j.neuroimage.2021.118424 (PMC8961003; doi:10.1016/j.neuroimage.2021.118424)
Supplement: Supplementary file 1 [file mmc1.pdf]

Supplementary Information

	N_b	L_b (μm)	Target D_s (μm)	Effective D_s (μm)	ν_s	Target D_b (μm)	Effective D_b (μm)	p_{ex} (%)
a) Overall cell diameter = 1000 μm	1	500	8	7.73	0.32	0.45	0.36	0.54
	1	500	12	11.60	0.32	0.83	0.66	0.81
	1	500	16	15.47	0.32	1.28	1.03	1.11
	1	500	20	19.36	0.32	1.78	1.44	1.38
	2	250	8	7.73	0.32	0.37	0.29	0.35
	2	250	12	11.59	0.31	0.67	0.55	0.56
	2	250	16	15.47	0.31	1.04	0.84	0.74
	2	250	20	19.34	0.31	1.46	1.19	0.95
	4	125	8	7.72	0.32	0.22	0.18	0.14
	4	125	12	11.58	0.32	0.41	0.34	0.22
	4	125	16	15.44	0.32	0.64	0.53	0.29
	4	125	20	19.32	0.31	0.90	0.75	0.38
	6	83	8	7.72	0.34	0.12	0.10	0.04
	6	83	12	11.58	0.34	0.23	0.19	0.07
	6	83	16	15.44	0.33	0.36	0.30	0.09
	6	83	20	19.30	0.33	0.51	0.43	0.12
b) Overall cell diameter = 400 μm	N_b	L_b (μm)	Target D_s (μm)	Effective D_s (μm)	ν_s	Target D_b (μm)	Effective D_b (μm)	p_{ex} (%)
	1	200	8	7.74	0.32	0.71	0.57	1.36
	1	200	12	11.62	0.32	1.31	1.06	2.08
	1	200	16	15.52	0.31	2.02	1.65	2.83
	1	200	20	19.42	0.31	2.82	2.32	3.57
	2	100	8	7.74	0.31	0.58	0.47	0.92
	2	100	12	11.61	0.31	1.07	0.88	1.44
	2	100	16	15.50	0.3	1.65	1.38	1.98
	2	100	20	19.39	0.3	2.30	1.95	2.53
	4	50	8	7.73	0.31	0.36	0.30	0.38
	4	50	12	11.59	0.3	0.66	0.56	0.58
	4	50	16	15.47	0.3	1.01	0.88	0.81
	4	50	20	19.34	0.29	1.42	1.26	1.06
	6	33	8	7.72	0.32	0.20	0.17	0.12
	6	33	121	11.58	0.31	0.37	0.33	0.2
	6	33	16	15.44	0.3	0.57	0.52	0.28
	6	33	20	19.32	0.29	0.80	0.74	0.37

Table S1 Parameters of the computational models of cellular meshes used in MC simulations.

S1. Additional simulation results

In the first part, we present additional simulation results for different cellular configurations and/or sequence parameters compared to the data shown in the main text which further support the results and discussion of this work.

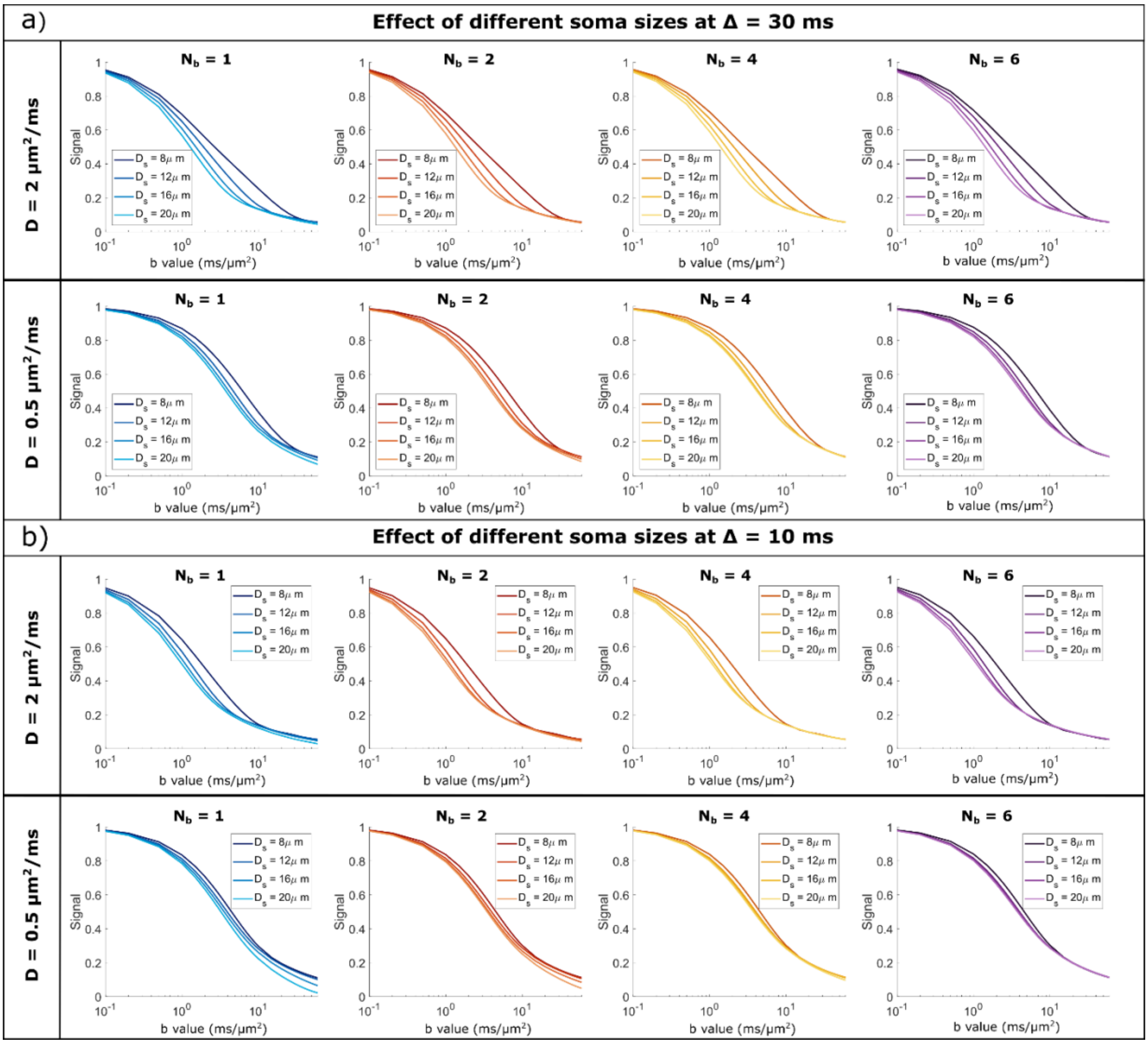

Figure S1 b-value dependence SDE signal for cells with different soma sizes and branching orders for a diffusion time of a) Δ = 30 ms and b) Δ = 10 ms.

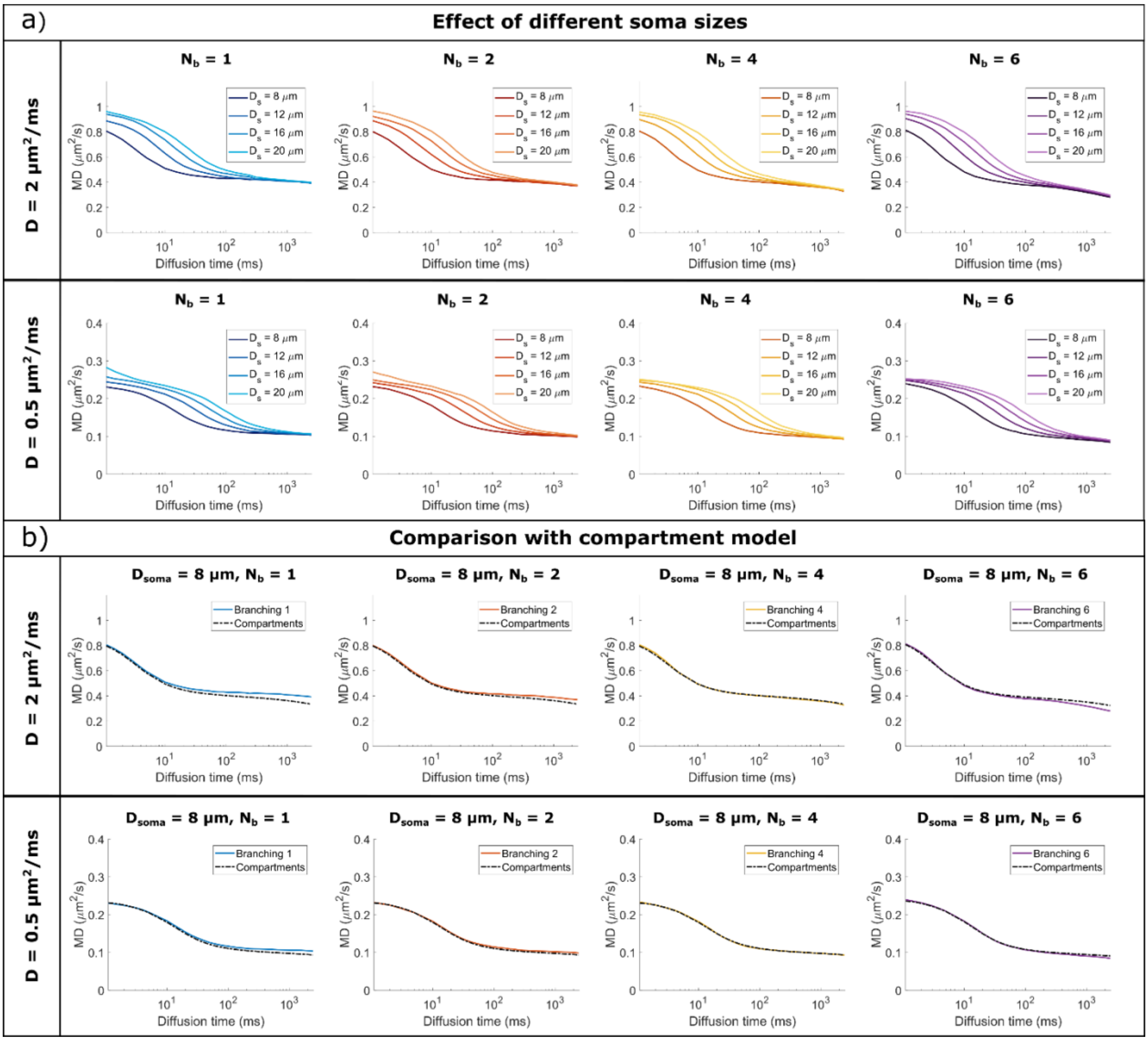

Figure S2 a) Effect of different soma sizes and branching orders on the MD time dependence for cells with a domain L = 1000 μm. b) Comparison of MD time dependence between simulated data and a two-compartment model for cells with different branching orders, D_s = 8 μm and L = 1000 μm.

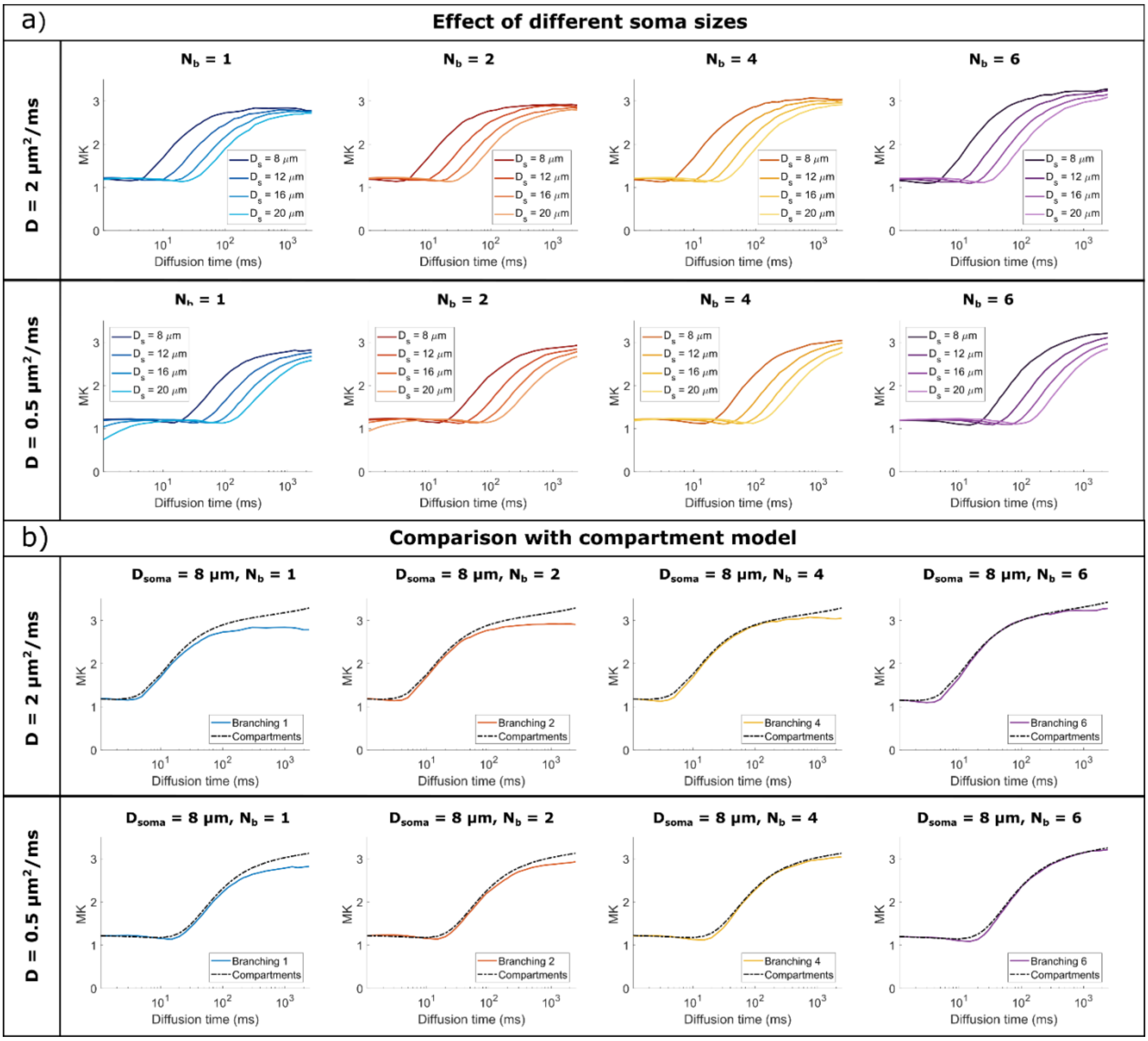

Figure S3 a) Effect of different soma sizes and branching orders on the MK time dependence for cells with a domain L = 1000 μm. b) Comparison of MK time dependence between simulated data and a two-compartment model for cells with different branching orders, D_s = 8 μm and L = 1000 μm.

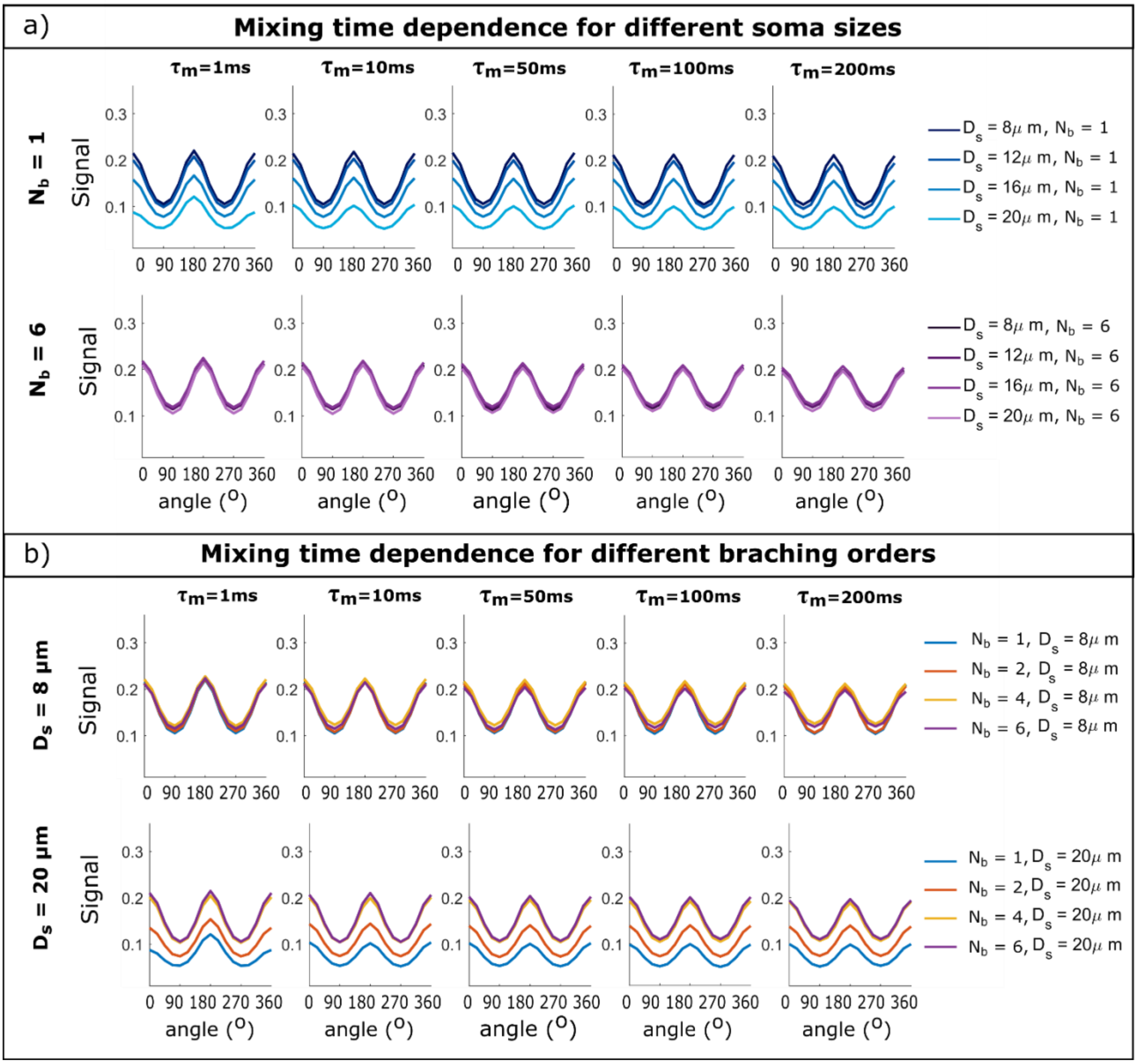

Figure S4. Dependence of DDE angular modulation on the mixing time for a) cells with different soma sizes and b) cells with different branching orders. The data is simulated for cells with a domain size of 400 μm, b-value of b = 16 ms/μm^2 and D = 0.5 μm^2/ms.

S2. Analysis of signal differences in the presence of noise

In this part, we present the impact of soma size and cell complexity on the SDE and DDE signal from the perspective of detectability at different noise levels. To this end, after the signal was averaged over the 10 cellular configurations, N_{noise} = 1000 instances of Gaussian noise with standard deviation $\sigma = 0.05$ (i.e. corresponding to an SNR of 20 in the b0 data) was added to each diffusion measurement. Then, the signal was averaged over directions, as described for each experiment. Following the directional average, the estimated metrics were computed.

For SDE sequences we investigate the signal differences as a function of b-value, as well as differences in MD and MK as a function of diffusion time, similar to the analysis presented in section 2.3, after noise was added to the data.

Figures S5a) and S6a) present the signal difference between configurations with the smallest diameter D_s = 8 μm and those with larger diameters, as a function of b-value for a diffusion time of 80 and 10 ms, respectively. Figures S5b) and S6b) present the signal difference between the MC simulations and the theoretical compartment model for cells of various soma size and branching orders, for a diffusion time of 80 and 10 ms, respectively.

Figures S7a) and S8a) present the MD and MK differences between configurations with the smallest diameter Ds = 8 μm and those with larger diameters, as a function of diffusion time, while Figures S7b) and S8b) present the MD and MK differences between the MC simulations and the theoretical compartment model, for the same parameters as the data presented in Figures 6 and 7.

For DDE sequences, we investigate the mixing time dependence of the amplitude of the signal modulation between measurements with parallel and orthogonal gradients. To calculate the amplitude modulation, we first compute the mean signal for measurements with parallel and anti-parallel gradients (i.e. measurements with $\varphi = 0$ and π in section 2.4.1) and then we subtract the mean signal for measurements with orthogonal gradients (i.e. measurements with $\varphi = \pi/2$ and $3\pi/2$ in section 2.4.1). To see whether changes are detectable, we analyse the difference in amplitude modulation between measurements with increasing mixing times and τ_m = 1 ms, in the presence of noise. Thus, after the signal was averaged over the 10 cellular configurations, we add N_{noise} = 1000 instances of Gaussian noise with standard deviation σ = 0.05 (i.e. corresponding to an SNR of 20 in the b0 data) to each diffusion measurement. Then, for each relative angle, the signal was averaged over the 8 different planes and the 5 in-plane rotations. After averaging, the amplitude modulation was computed as described above.

Figure S9a) presents the difference in amplitude modulation between measurements with increasing mixing time and τ_m = 1 ms for cells with different soma diameters and branching orders. Figure S9b) illustrates the difference in amplitude modulation between the MC simulations and the theoretical compartment model for cells with different soma diameters and branching orders.

To further investigate the effect of noise on the mixing time dependence of the estimated apparent microscopic anisotropy, we add N_{noise} = 1000 instances of Gaussian noise with standard deviation σ = 0.05 (i.e. corresponding to an SNR of 20 in the b0 data) to each diffusion measurement from the 5-design protocol employed in section 2.5.2. Figure S10 illustrates the difference in apparent μA between the MC simulations and the theoretical compartment model as a function of mixing time for cells with different soma diameters and branching orders, for DDE sequences with Δ = 5 ms, and Δ = 30 ms.

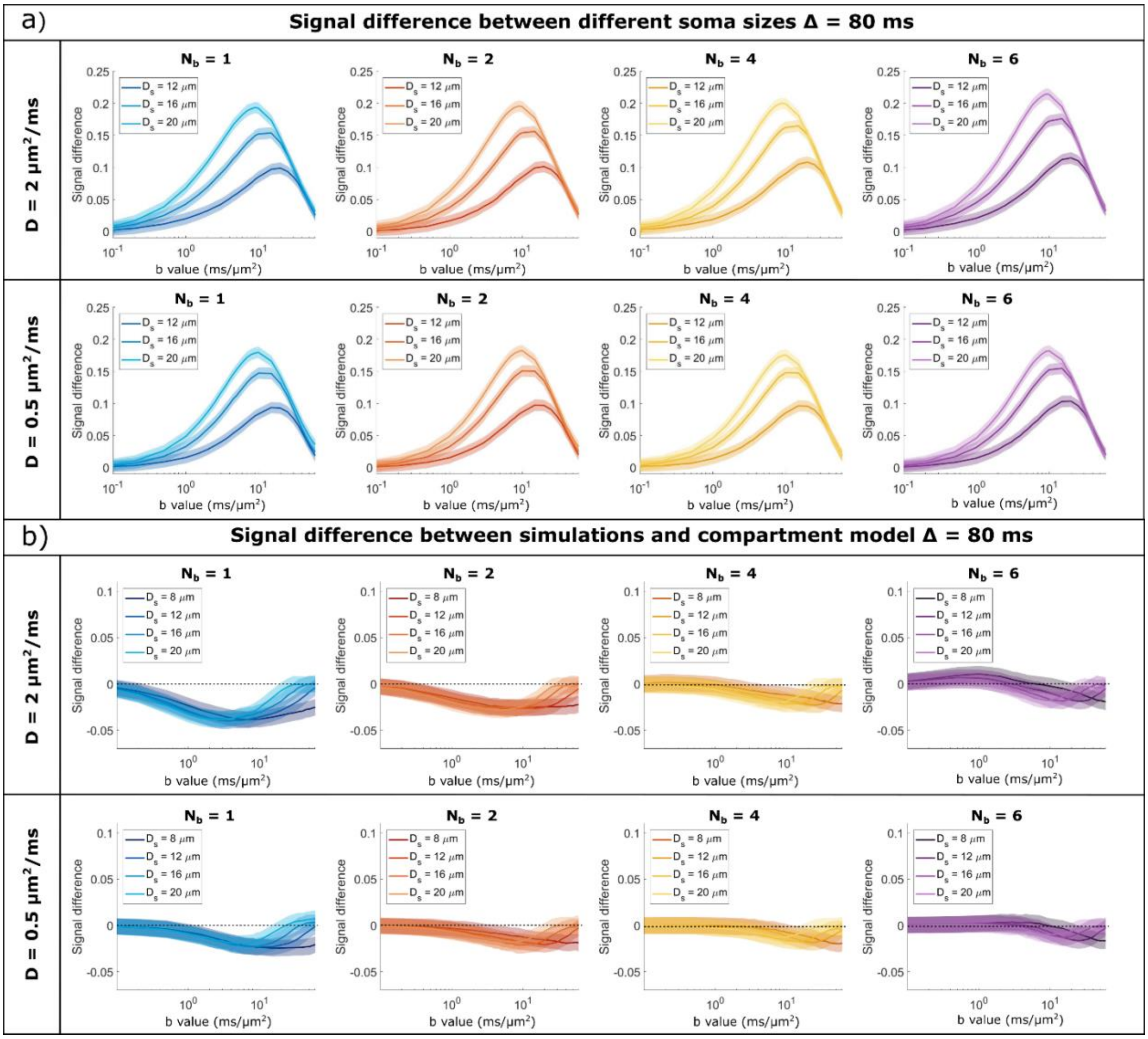

Figure S5 a) Signal difference between cells with larger soma diameters (12 – 20 μm) and cells with Ds = 8 μm as a function of b-value, for cells with Nb = {1, 2, 4, 6} and L = 400 μm. b) Signal difference between the MC simulations and the compartment model for cells with different soma diameters and branching orders. The shaded area represents the standard deviation over 1000 noisy datapoints. When the shaded areas do not overlap, the differences are detectable, meaning that they are statistically significant with a p<0.01. The data is simulated at Δ = 80 ms.

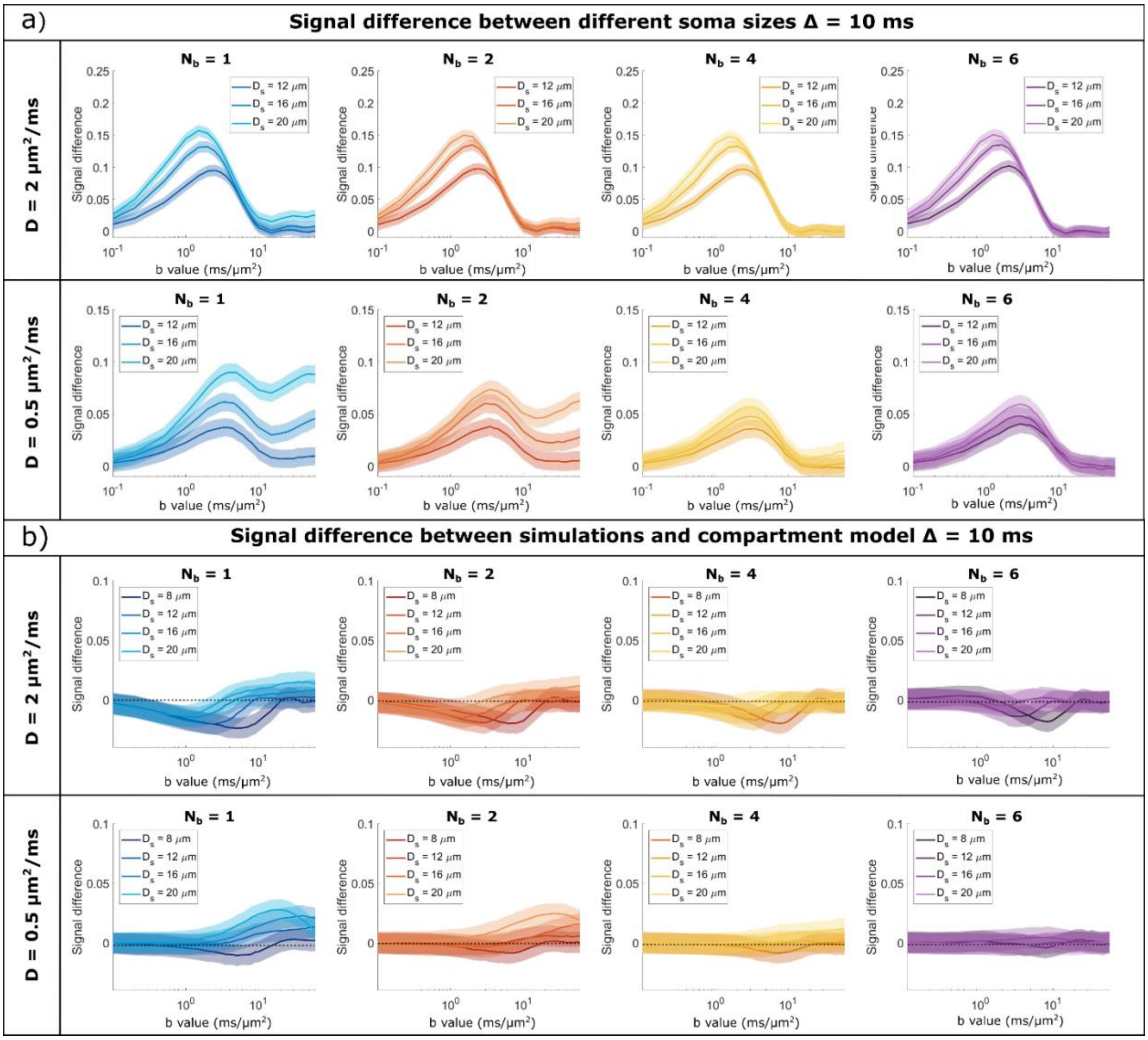

Figure S6 a) Signal difference between cells with the smallest diameter Ds = 8 μm and those with larger diameters (12 – 20 μm) as a function of b-value, for cells with Nb = {1, 2, 4, 6} and L = 400 μm. b) Signal difference between the MC simulations and the compartment model for cells with different soma diameters and branching orders. The shaded area represents the standard deviation over 1000 noisy datapoints. When the shaded areas do not overlap, the differences are detectable, meaning that they are statistically significant with a p<0.01. The data is simulated at Δ = 10 ms.

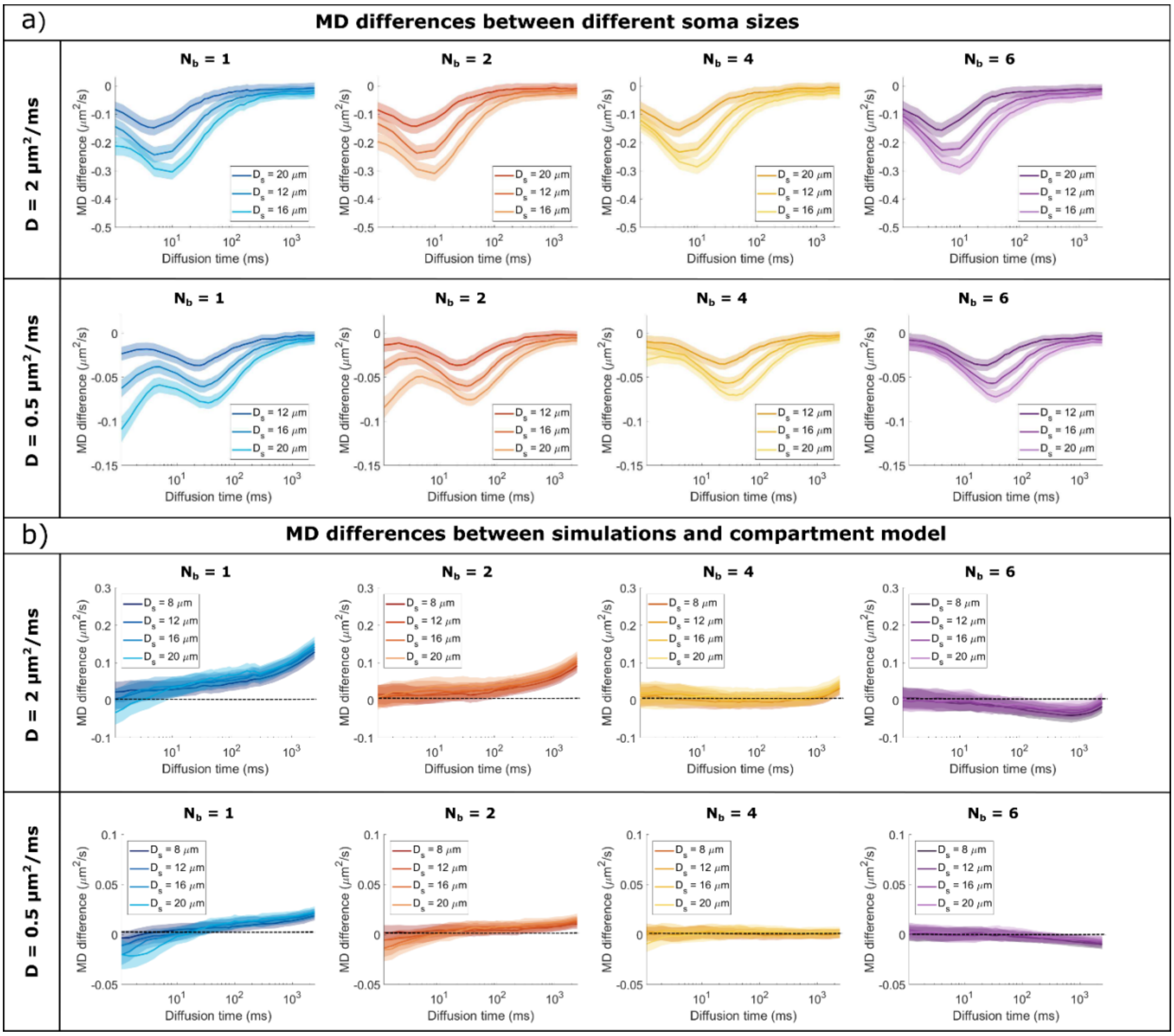

Figure S7 a) MD differences between cells with the smallest diameter Ds = 8 µm and those with larger diameters (12 – 20 µm) as a function of diffusion time, for cells with Nb = {1, 2, 4, 6}[1] and L = 400 µm. b) MD differences between the MC simulations and the compartment model for cells with different soma diameters and branching orders. The shaded area represents the standard deviation over 1000 noisy datapoints. When the shaded areas do not overlap, the differences are detectable, meaning that they are statistically significant with a p<0.01.

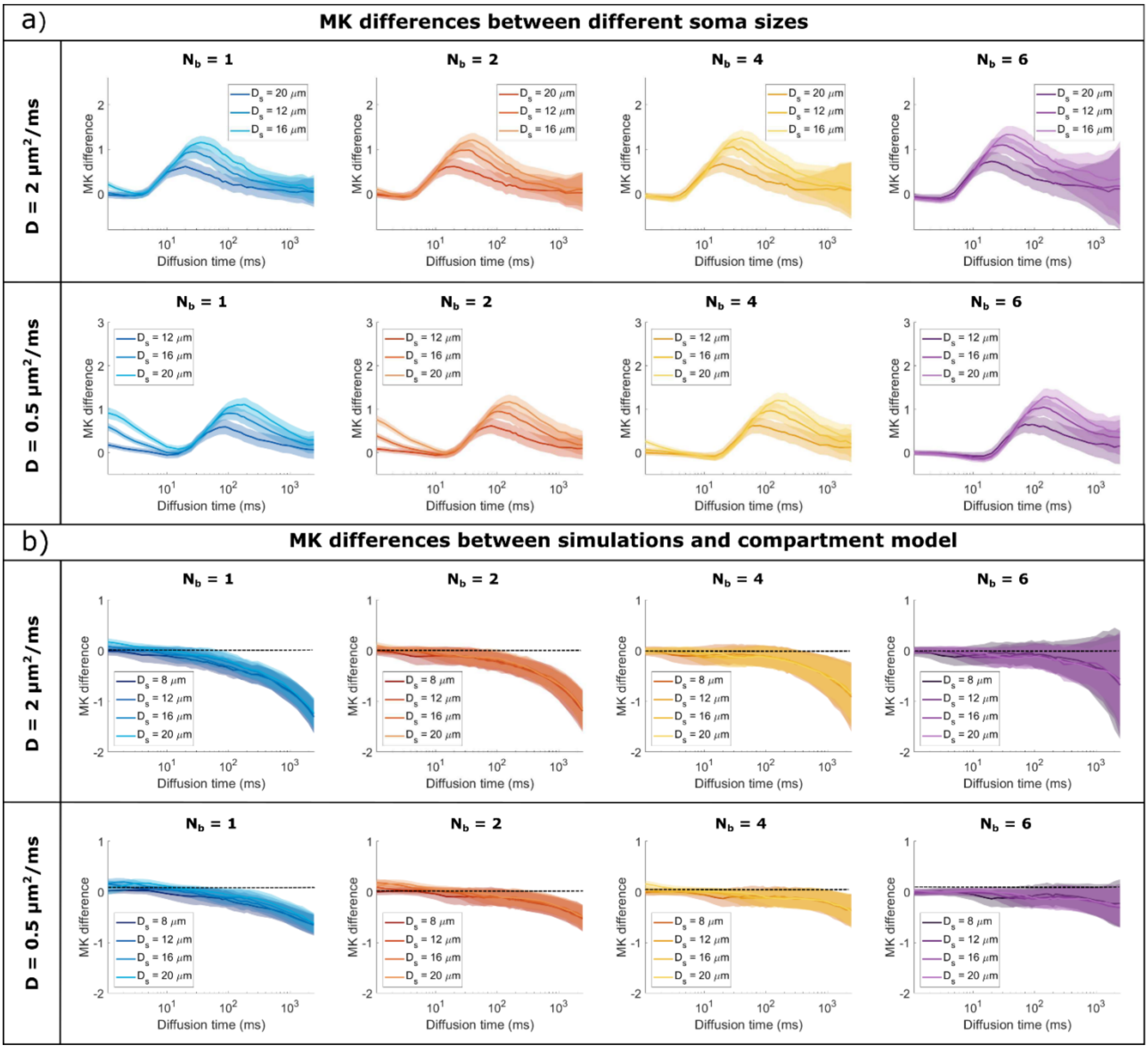

Figure S8 a) MK differences between cells with the smallest diameter Ds = 8 µm and those with larger diameters (12 – 20 µm) as a function of diffusion time, for cells with Nb = {1, 2, 4, 6}[1] and L = 400 µm. b) MK differences between the MC simulations and the compartment model for cells with different soma diameters and branching orders. The shaded area represents the standard deviation over 1000 noisy datapoints. When the shaded areas do not overlap, the differences are detectable, meaning that they are statistically significant with a p<0.01.

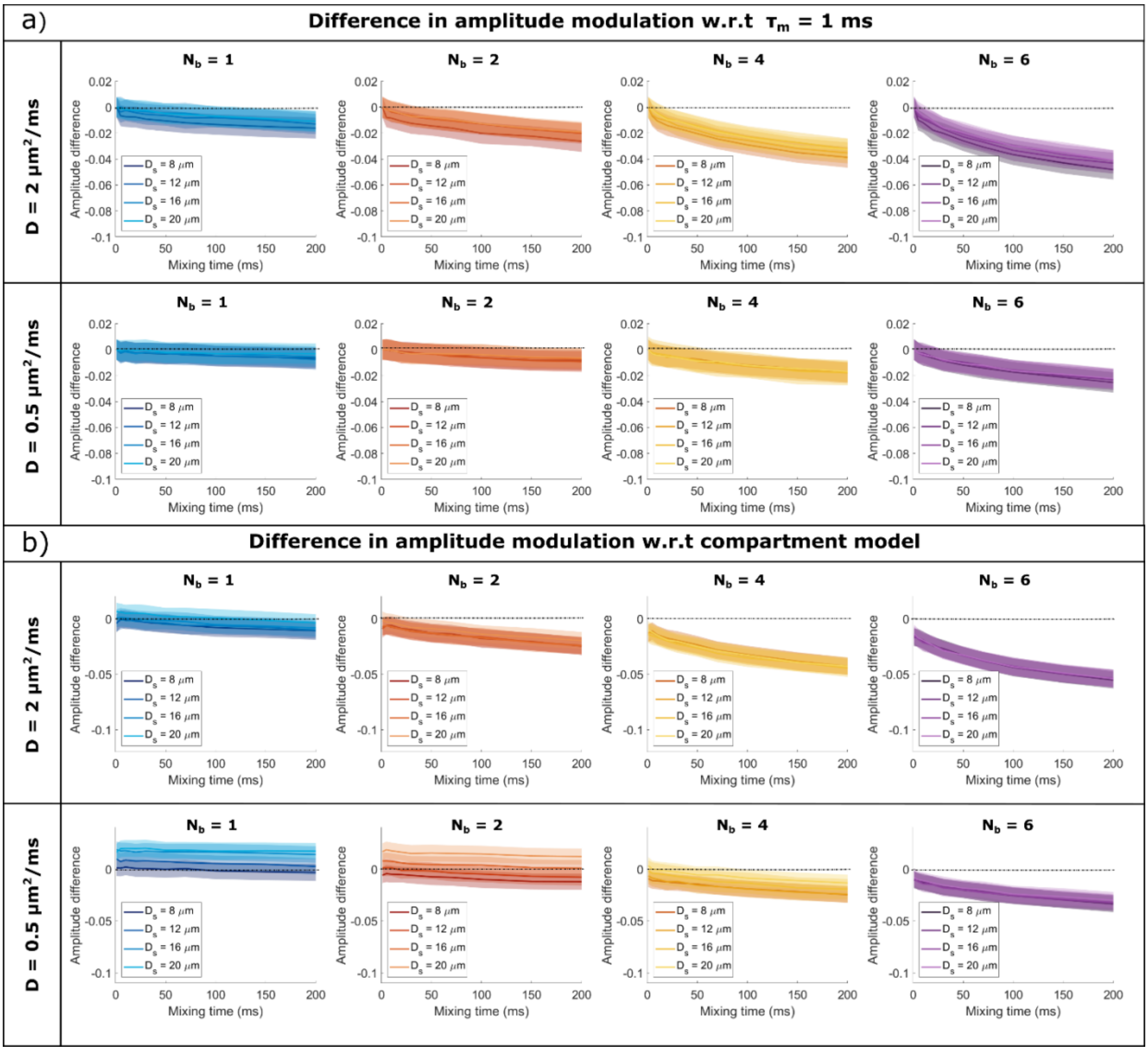

Figure S9a) Difference in amplitude modulation between measurements with increasing mixing time and τ_m = 1 ms for cells with different soma diameters and branching orders. b) difference in amplitude modulation between the MC simulations and the theoretical compartment model for cells with different soma diameters and branching orders. The simulations have been performed with the same parameters as detailed in Section 2.4.1 (Δ = 5 ms, b = 4 ms/μm^2 for D = 2 μm^2/ms and b = 16 ms/μm^2 for D = 0.5 μm^2/ms). The shaded area represents the standard deviation over 1000 noisy datapoints. When the shaded areas do not overlap, the differences are detectable, meaning that they are statistically significant with a $p<0.01$.

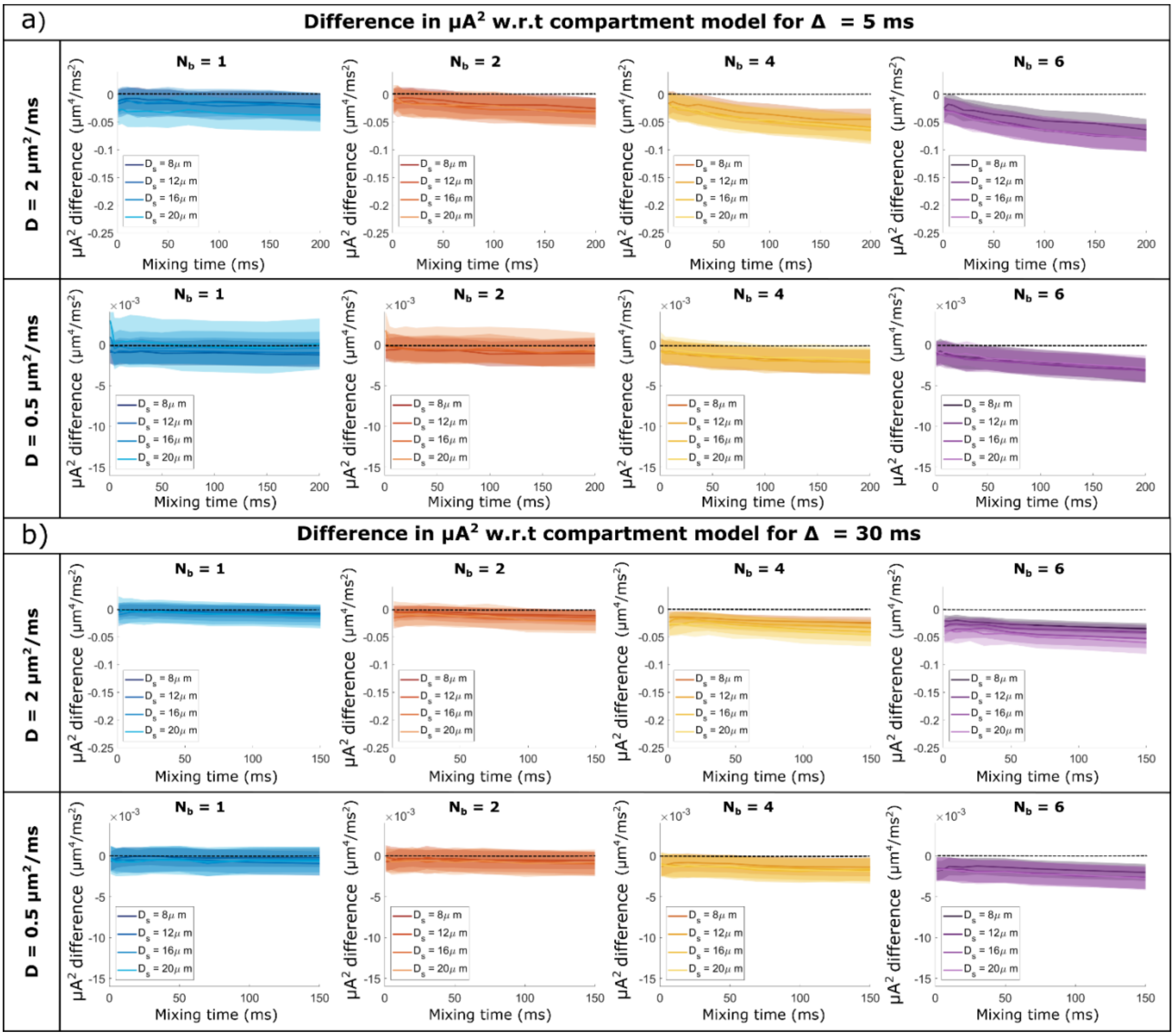

Figure S10a) Difference in μA^2between the MC simulations and the theoretical compartment model for cells with different soma diameters and branching orders for DDE with $\Delta = 5$ ms. The simulations have been performed with the same parameters as detailed in Section 2.5.2 ($b = 4$ ms/μm^2 for $D = 2$ μm^2/ms and $b = 16$ ms/μm^2 for $D = 0.5$ μm^2/ms) . S10b) Same as a), just for $\Delta = 30$ ms. The shaded areas represent the standard deviation of the estimated metrics over 1000 instances of noise. When the shaded areas do not overlap, the differences are detectable, meaning that they are statistically significant with a $p<0.01$.

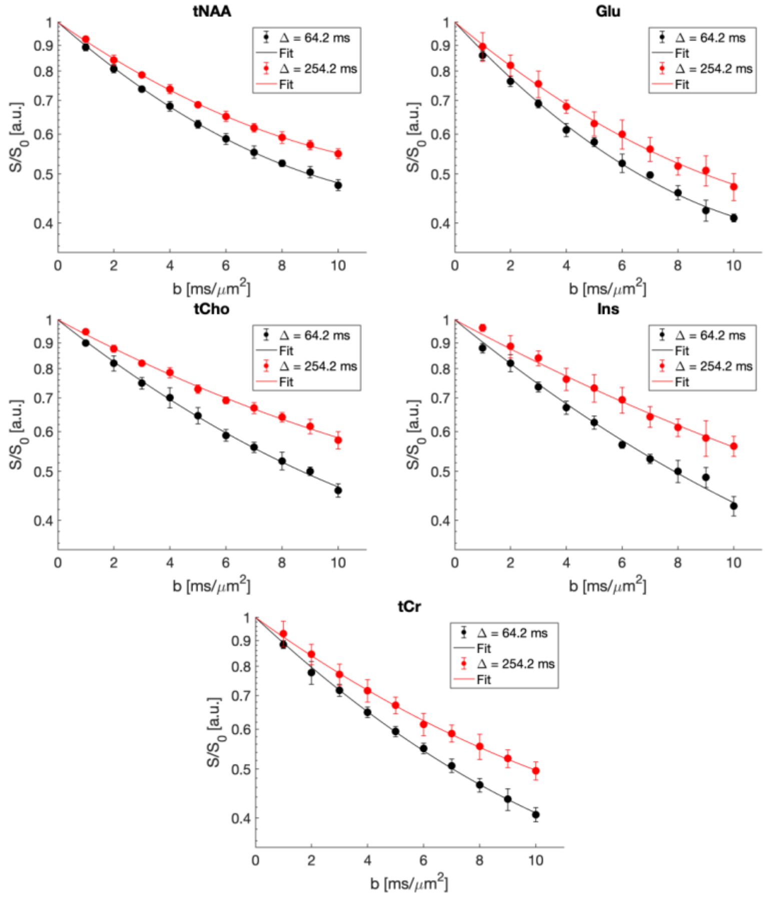

Metabolite	Δ (ms)	MD (μm²/ms)	MK
tNAA	64.2	0.112 [0.109, 0.115]	1.851 [1.769, 1.933]
	254.2	0.089 [0.087, 0.092]	2.220 [2.112, 2.329]
Glu	64.2	0.137 [0.128, 0.145]	1.539 [1.384, 1.695]
	254.2	0.106 [0.101, 0.112]	1.714 [1.508, 1.921]
tCr	64.2	0.120 [0.115, 0.125]	1.276 [1.114, 1.438]
	254.2	0.092 [0.087, 0.096]	1.567 [1.326, 1.808]
tCho	64.2	0.101 [0.096, 0.106]	1.442 [1.208, 1.677]
	254.2	0.068 [0.063, 0.073]	1.847 [1.314, 2.379]
Ins	64.2	0.104 [0.100, 0.109]	1.141 [1.060, 1.223]
	254.2	0.069 [0.065, 0.072]	1.327 [1.172, 1.482]

Figure S11. dMRS data from [5] analysed using the cumulant expansion of the diffusion weighted signal up to the second order. The datapoints are average signal values over four mice and the error bars are the corresponding standard deviations. The solid lines are the fit of the second-order cumulant expansion representation (i.e., Eq.[4] in the main text) to the data. The estimated MD and MK values for each diffusion gradient separation Δ are reported in the table, with the 95% confidence interval reported in square brackets. More information about the data acquisition and processing can be found in [5].

S3. Quantification of exchange effects from the AXR model

In this analysis we aim to quantify the impact of exchange between two compartments on the DDE signal using the simple apparent exchange rate (AXR) model. Following equations 6 and 7 in [2] we can calculate the DDE signal as a function of b-value and mixing time as:

$$S(b, \tau_m) = S_0(\tau_m)\, exp(-b\, ADC'(\tau_m)),$$

where $S_0(\tau_m)$ is the signal at b = 0 and accounts for the effects of longitudinal relaxation during the mixing time and the apparent diffusion coefficient $ADC'(\tau_m)$, in the limit $b \to 0$, is determined by the apparent diffusivity and exchange rate of the two compartments:

$$ADC'(\tau_m) = ADC(1 - \sigma\, exp(-\tau_m AXR)),$$

where $ADC = f_1^e D_1 + (1 - f_1^e) D_2$ is the equilibrium apparent diffusion coefficient of the system, σ is the filter efficiency and AXR is the apparent exchange rate. From previous literature the estimated parameter values (ADC, σ and AXR) depend on the tissue [1, 3, 4], but also on the b-value of the first gradient pair, with [4] reporting a drastic decrease in AXR between values estimated with a filter of b = 250 s/mm^2 and b = 900 s/mm^2. Reported ADC values were between ~ 0.6 to 0.8 for WM and ~

0.8 and 1 in GM [1, 3] [4]; σ values estimated between ~0.2 and 0.3 in both WM and GM; AXR values were between 0.4 and 0.8 in GM and between 0.6 and 0.9 in WM, for a filter b-value of 900 s/mm^2.

To estimate the impact on the DDE sequences simulated in this work, we calculate the DDE signal difference between measurements with τ_m = 1 ms and τ_m = 200 ms for two combinations of parameter values measured in GM: σ = 0.23, ADC = 0.75 mm^2/s and AXR = 0.72 s^{-1} from [4] and σ = 0.23, ADC = 0.95 mm^2/s and AXR = 0.4 s^{-1} from [1]. Assuming $S_0(\tau_m)$ is constant and the same parameters apply for a filter with b = 2000 s/mm^2, we obtain normalized signal differences due to exchange effects of 0.009 and 0.004, respectively. These differences might become even smaller given the decrease in AXR which has been reported when the filter was increased from 250 to 900 s/mm^2. Even with the current values the signal differences due to exchange as modelled by AXR are >5 times smaller than the differences quantified in Figure S7 due to increasing the branching order.

1. Nilsson, M., et al., *Noninvasive mapping of water diffusional exchange in the human brain using filter-exchange imaging.* Magn Reson Med, 2013. **69**: p. 1572-1580.
2. Lasic, S., et al., *Apparent Exchange Rate Mapping with Diffusion MRI.* Magn Reson Med, 2011. **66**: p. 356-365.
3. Lampinen, B., et al., *Optimal Experimental Design for Filter Exchange Imaging: Apparent Exchange Rate Measurements in the Healthy Brain and in Intracranial Tumors.* Magn Reson Med, 2017. **77**(3): p. 1104-1114.
4. Bai, R., et al., *Feasibility of filter-exchange imaging (FEXI) in measuring different exchange processes in human brain.* NeuroImage, 2020. **219**: p. 117039.
5. Ligneul, C., Palombo, M., and Valette, J. *Metabolite diffusion up to very high b in the mouse brain in vivo: Revisiting the potential correlation between relaxation and diffusion properties*. Magn Reson Med, 2017. **77**(4), 1390-1398.
